# East Anglian Sanatorium for Consumptives at Broomfields, Nayland, Suffolk

**Published:** 1901-12-14

**Authors:** 


### The Institutional Workshop.

### EAST ANGLIAN SANATORIUM FOR CONSUMPTIVES AT BROOMFIELDS, NAYLAND, SUFFOLK.

To begin with, this sanatorium has the advantage, hardly to be over-estimated, of having been built specially for the treatment of phthisis, and, further, it has been very well designed and equally well carried out, we believe, under the immediate supervision of Dr. Jane Walker, of Gower Street, London. The plan is that of a much expanded broad arrow, the shaft being short and containing the hall, lady superintendent's room, visitors' room, servants' hall, etc., and at right angles to the shaft and to the north are the billiard-room, dining-hall, kitchen, and kitchen offices. The dining-hall is a most beautiful and cheerful-looking room, and in spite of this it is admirably adapted for its purpose, the beautiful and the useful having been both secured in its construction. The expanded barbs of the arrow face almost South. The centre is given up, on ground and first floors, to spacious verandahs and balconies; the entrances to these being so arranged that deck-chairs or bedsteads can be easily wheeled through them. On either side of these verandahs are the patients' rooms; and northwards run the communicating corridors. These corridors are about six feet wide, are well lighted, and are floored with teak. By this simple form of construction the hospital proper is only one room thick, hence the cross ventilation is easily and efficiently carried out. There are rooms for 35 patients. The sanitary blocks project from the North side of the communicating corridors and they are correctly designed, being separated from the main building by short cross-ventilated passages.

The estate consists of upwards of 90 acres. These extensive grounds are quite private, and abound in delightful walks, while the balconies and verandahs command views many miles in circuit.

The whole building is lighted by the electric light and warmed by open fireplaces and low-pressure steam coils. Electric bells are fitted up between the patients' rooms and the nurses' rooms, and the laboratory is equipped with electric lamps for throat examination. In short every point essential for the modern open-air treatment of consumption has been carried out with skill and with the forethought born of experience.

The institution is visited twice a week by Dr. Jane Walker, and Dr. M. Paine is the resident physician. The usual *régime* of the sanatorium is as follows:—Before breakfast the resident physician visits all patients, and breakfast is served at eight o'clock. Patients, except those not allowed for medical reasons, take their prescribed amount of exercise between breakfast and midday when they all rest until 1 o'clock, the dinner hour. After dinner there is more regulated exercise. Tea is served at 4 o'clock. Then more rest until supper at 7 o'clock. The temperatures and pulses of all the patients are taken three times a day; and patients are regularly seen by the physician before dinner and supper, and again at bedtime if necessary. The patients are weighed weekly, and it would seem that between the day of admission and the day of discharge there is an average gain in weight of 28 pounds. There is evidently a strong medical element (we do not mean medicinal) in the treatment of patients in this sanatorium; and beyond doubt this is the correct principle. The duration of the treatment is from three to six months. The former period may occasionally prove sufficient; but every consumptive patient, and above all, every consumptive patient's guardian, ought to bear in mind that although recovery may seem to have taken place, complete stability is unlikely so soon, and that at least six months should be devoted to the cure. In her able little pamphlet, Dr. Jane Walker remarks that treatment is directed "not only to the arrest of the disease, but to the permanent raising of the standard of the patient's health in order that recurrent attacks may be warded off." No patient should forget these words, and every physician should remember another line of Dr. Walker's that "no two cases can be treated on exactly the same lines; we must consider not only the individual sickness, but the sick individual."

As to the proportion of recoveries, it is very difficult from the data before us to arrive at any very definite conclusion; but it may fairly be assumed that in the first stage of the disease the majority, perhaps the large majority, will recover under suitable treatment in these sanatoria. In the second stage the proportion will be much lower, and in the third very much lower than in the second. Dr. Jane Walker summarises her own cases as follows:—Total number 177, recovered 45, improving 54, going back 13, lost sight of 9, still under treatment 29, died 27. In these statistics the various stages are not given. A word as to the means of reaching the Nayland Sanatorium: From Liverpool Street to Bures, changing carriages at Marks Tey, takes about an hour and a half. At Bures waggonettes are generally waiting at the station, or carriages of any kind may be hired at the Eight Bells Hotel. The distance to the sanatorium is about three miles, and he who has preconceived ideas of the flatness of the country, or thinks Suffolk an uninteresting county, will be surprised at the beautifully undulating scenery which opens before him, and at the Devonshire-like half-mile of lane ending the drive as he turns into Broomfields, which claims to be "the sunniest spot in England."

